# Adjuvant-driven epitope hierarchy correlates with the protective efficacy of FimA vaccine against *Klebsiella pneumoniae*

**DOI:** 10.3389/fimmu.2026.1796753

**Published:** 2026-06-22

**Authors:** Guangyang Ming, Longlong Chen, Pengju Yan, Xiaoqiong Wang, Zhifu Chen, Qiang Gou, Yue Yuan, Jiayi Chen, Haiming Jing, Ping Luo, Quanming Zou, Jinyong Zhang, Zhiyong Liu, Renjian Hu, Zhuo Zhao

**Affiliations:** 1 National Engineering Research Center of Immunological Products, Department of Microbiology and Biochemical Pharmacy, College of Pharmacy and Laboratory Medicine, Army Medical University, Chongqing, China; 2 Chongqing Huazhi Biopharmaceutical Co., Ltd., Chongqing, China; 3 Department of Laboratory Medicine, Southwest Hospital, Third Military Medical University (Army Medical University), Chongqing, China; 4 School of Pharmacy and Bioengineering, Chongqing University of Technology, Chongqing, China

**Keywords:** adjuvants, FimA, immunodominant epitope, Klebsiella pneumoniae, Pneumonia

## Abstract

Hypervirulent *Klebsiella pneumoniae* (hvKP) variants represent urgent multidrug-resistant threats against which no licensed vaccine exists. FimA is indispensable for KP mucosal colonization and is thus a promising vaccine target; however, its protective immunodominant B-cell epitopes in vaccines with different adjuvants against KP remain undefined. Here, we revealed how adjuvants shape different hierarchies of the immunodominant B-cell epitopes of FimA and studied the humoral anti-FimA response and protective efficacy in a murine model of acute pneumonia. BALB/c mice were intramuscularly immunized with recombinant FimA formulated in three clinically trialed adjuvants: AddaVax, AddaS03, and AlPO_4_, whereafter they were challenged with lethal doses of the hvKP YBQ strain. Under the experimental conditions used in this immunization-challenge model, AddaS03-immunized mice exhibited 100% survival and reduced pulmonary bacterial load, whereas AddaVax-immunized mice showed only 40% survival. Furthermore, FimA-immunized groups with different adjuvants exhibited different opsonophagocytic killing activities and inflammatory cytokine levels, likely explaining the variability in protective immunity. ELISA-based linear B-cell epitope mapping of FimA with different adjuvants revealed three novel immunodominant epitopes—their hierarchies were altered by different adjuvants. Further, a causal link might exist between FimA epitope hierarchy and protective efficacy in hvKP-infected mice. Immunization with a mixture of these FimA immunodominant epitopes plus AlPO_4_ achieved an 80% protection rate, where it exerted a potent therapeutic effect in combination with low-dose meropenem. Our findings revealed that adjuvants enhance anti-FimA immunity and modulate FimA epitope hierarchies across different vaccinated groups. Therefore, rational adjuvant selection may elicit effective FimA-induced responses in KP vaccines, wherein different FimA immunodominant epitopes induce protective humoral immune responses.

## Introduction

*Klebsiella pneumoniae* (KP) is a life-threatening pathogen responsible for hospital-acquired infections, including respiratory tract, bloodstream, and urinary tract infections (1). It accounts for approximately one-third of all Gram-negative bacterial infections in hospitalized patients (2). Due to its rising prevalence and multidrug resistance, KP poses a significant threat to global public health (3); the 2024 World Health Organization Bacterial Priority Pathogens List named carbapenem-resistant KP (CRKP) as a top-priority pathogen (84% total score) owing to its substantial public health burden (4). Based on its virulence and pathogenic characteristics, KP can be classified into two major types: classic KP and hypervirulent KP (hvKP) (5). Notably, the clinical landscape has become more complex with the emergence of CRKP strains that acquire hypervirulent phenotypic and genetic traits, which are often confused with hypervirulent KP strains that gain multidrug resistance genetic elements. To distinguish these two distinct yet clinically threatening lineages, some authors have proposed a novel classification system designating them as ultravirulent KP (Uv-KP) and supervirulent KP (Sv-KP), respectively (6, 7). Of 124 countries worldwide, 43 have reported the presence of hvKP, and 12 have specifically reported prevalence of the hvKP ST23-KL1 strain (8). Globally, the prevalence of CRKP has been reported to be high in Greece and South Asia and relatively lower in high-income regions of North America (9, 10). The serotypes of multidrug-resistant hvKP (MDR-hvKP) vary according to sequence type (ST) and geographic region. For instance, serotypes K64 and K47, associated with the KP ST11 strain, are the most commonly reported among MDR-hvKP isolates in China (11).

The escalating prevalence of KP infections and increasing antibiotic resistance underscore the need to develop a prophylactic vaccine as a promising intervention strategy (12). Consequently, the identification of key antigens that elicit a robust immune response is a critical determinant of effective vaccine design. The pilus system plays a critical role among the virulence factors implicated in KP pathogenesis (13). As the principal structural subunit of type 1 pili, FimA accounts for >95% of the total pilus protein and, in conjunction with the FimH adhesin, constitutes the complete pilus assembly (14, 15). The functional significance of this protein lies in its ability to polymerize into adhesive pili, thereby mediating bacterial attachment to host cell surfaces. Therefore, FimA is a promising antigenic target for vaccine development. This premise is supported by prior experimental evidence; for instance, a trivalent KP vaccine containing FimA, among other antigens, conferred significant protection in a murine pneumonia model (16). Furthermore, immunization with this vaccine formulation elicited a potent humoral immune response characterized by a significant increase in IgG, IgG1, and IgG2a antibody levels (17). Collectively, these findings substantiate the inclusion of FimA as a promising candidate antigen for future KP vaccine development.

The critical role of antibody responses in conferring protection against KP is well-established (17). Accordingly, the identification of immunodominant epitopes within target antigens may provide useful information for vaccine design, as these determinants can represent major targets of antibody responses (18).​ Notably, immunodominance hierarchies are not fixed properties of the antigen but can be substantially reshaped by the adjuvant used in vaccine formulations (19). Such adjuvant-associated modulation may contribute to differences in the composition and functional characteristics of the antibody repertoire. Based on these insights, we propose that the adjuvant-driven reprogramming of B-cell immunodominance may serve as a tunable strategy to enhance the protective efficacy of FimA, effectively improving its immunogenicity. Therefore, the deliberate steering of immune recognition toward specific protective epitopes should be regarded as an essential design principle for the development of next-generation KP vaccines.

Adjuvants are essential components in vaccine development against KP because they enhance the immunogenicity of antigenic targets, such as FimA, by modulating both innate and adaptive immune pathways (20). AddaS03, a squalene-based oil-in-water emulsion analogous to the licensed AS03 adjuvant, enables antigen dose-sparing and promotes a balanced Th1/Th2 immune response. Clinical studies have shown that it induces robust early myeloid and lymphoid activation after vaccination (21). AddaVax, another squalene-based emulsion similar to MF59, recruits and activates antigen-presenting cells and stimulates cytokine secretion, thereby supporting either Th1 or Th2-polarized immunity (22). Notably, both AS03 (the reference for AddaS03 used in GSK vaccines) and MF59 (the reference for AddaVax used in Seqirus vaccines) have been approved for human use in pandemic influenza vaccines (23). Aluminum phosphate (AlPO_4_), a widely employed inorganic adjuvant in human vaccines, functions through antigen adsorption, depot formation, and innate immune pathway activation (24), and it has consistently induced strong antibody responses in bacterial vaccine models (25). Based on their complementary immunomodulatory properties and established safety profiles, these adjuvants were chosen to comprehensively compare their capacity for enhancing the protective immunity elicited by FimA against hvKP challenge.

In this study, we evaluated the protective efficacy of a recombinant FimA vaccine with three adjuvants that have been used in clinical trials, against the hvKP YBQ strain in a murine model of acute pneumonia following intramuscular immunization. The FimA–adjuvant vaccine demonstrated robust protective potential. Our findings confirm the robust protective potential of the FimA–adjuvant vaccine and reveal that adjuvants play a critical role in modulating FimA-targeted immunity, including reshaping the immunodominance hierarchy of B-cell epitopes associated with FimA. Additionally, preliminary validation suggests that epitope-based vaccines derived from these findings may hold promise for the prevention and treatment of hvKP infections. Collectively, our work provides a useful basis for the development of advanced FimA-based vaccines against hvKP, which highlights the potential association between adjuvant selection and FimA-mediated immune response profiles.

## Materials and methods

### Ethics statement

All animal experiments were approved by the Animal Ethical and Experimental Committee of the Army Medical University (Chongqing, China; Permit AMUWEC20230478) and performed in accordance with their rules and regulations.

### Animals and antigens

Specific pathogen-free female BALB/c mice (6–8 weeks old) were purchased from Sichuan Vital River Laboratory Animal Technology Co., Ltd. (Sichuan, China). The FimA antigen was expressed and purified as follows.

The recombinant full-length FimA protein was prepared using genetic recombination techniques, following a comprehensive analysis of the gene sequences of KP strains through bioinformatics methods. Gene and amino acid information for the FimA antigen was obtained from NCBI (Sequence ID: WP_032434944.1). Specific primers were designed to clone the target gene of FimA, which was digested with BamHI and XhoI and subsequently ligated into the pGEX-6P-1 plasmid. *Escherichia coli* was used as the host cell to construct the recombinant strain. Target protein expression was induced with a GST tag, followed by enzymatic cleavage to remove the GST tag. The recombinant FimA protein lacking the GST tag had a molecular weight of 17.3 kDa. The purified protein was further analyzed via sodium dodecyl sulphate-polyacrylamide gel electrophoresis to confirm its molecular weight, and a FimA protein with >95% purity was successfully prepared.

### Peptide synthesis and KLH conjugations

The 18-mer peptides with a length of 12 amino acids overlapping to cover the full length of FimA (Sequence ID: WP_032434944.1) were synthesized and purified by GL Biochem Ltd. (Shanghai, China). The OVA_192–201_ (EDTQAMPFRV) peptide was used as a negative control. The purity of these peptides was expected to be ≥95%. The peptides were dissolved in dimethyl sulfoxide at a concentration of 0.5 mg/mL and stored at −80 °C before use. For each immunodominant peptide, peptide–KLH (Keyhole limpet hemocyanin) conjugation was performed by GL Biochem Ltd.

### Immunization and infection

To determine the immunogenicity of FimA or the mixture of FimA immunodominant epitope–KLH (Mix-peptides), mice were randomized into different groups (n = 10) and intramuscularly immunized with 50 μg FimA combined with 50 μL AddaVax (InvivoGen, San Diego, CA, USA), AddaS03 (InvivoGen), or AlPO_4_ (InvivoGen) in a total volume of 100 μL with adjuvant or phosphate-buffered saline (PBS) only on days 0, 7, and 14. One week after the last booster, antisera from each group were collected for subsequent experiments. To determine the protective efficacies of the immunodominant epitopes, mice (n = 10) were intramuscularly immunized with 100 μg of each epitope–KLH conjugate combined with 100 μL AlPO_4_ on days 0, 7, and 14. The Mix-peptides included 33 μg of each epitope–KLH conjugate. One week after the final booster, mice were infected with 8 × 10^6^ colony-forming units (CFU) of the hvKP YBQ strain in 100 mL saline, and the survival rates in each group were monitored for seven days following infection.

The hvKP YBQ strain was obtained from the Department of Clinical Laboratory at the Southwest Hospital, Army Medical University. Detailed information on YBQ can be found in literature (26). YBQ was identified as an hvKP strain based on string testing, gene sequencing, and animal challenge experiments; it demonstrated significantly higher virulence than that of the reference KP ATCC 700721 strain (purchased from the American Type Culture Collection, Manassas, VA, USA).

### Bacterial burden and tissue histology

Mice in each group were infected with 1 × 10^6^ CFU hvKP YBQ seven days after the final immunization (n = 8), and their lungs harvested 48 h after infection. Bacterial burden in the organs was quantified by preparing organ homogenates in PBS and plating fivefold serial dilutions onto Luria–Bertani (LB) plates. The colonies were counted after 24 h of incubation at 37 °C. The number of CFU/tissue was calculated from each plate.

For histopathological assessment, the organs were preserved in 4% paraformaldehyde and processed into paraffin-embedded blocks. Sections with a thickness of 4 μm were prepared and subjected to hematoxylin and eosin (H&E) staining, followed by microscopic evaluation.

### Immunoglobulin subtyping

One week after the final immunization, the mice were exsanguinated, and serum samples collected thereafter. FimA-specific IgG titers were determined using ELISA. In brief, the wells of microtiter plates (Corning Inc., Corning, NY, USA) were coated with FimA (0.5 µg/well) in 50 mM carbonate buffer (pH 9.5) at 4 °C overnight. Non-specific binding was blocked with 2% (v/v) bovine serum albumin (BSA) at 37 °C for 2 h. Serum samples were serially diluted twofold in PBS (starting at 1:100) and used as the primary antibodies. The secondary antibodies comprised horseradish peroxidase (HRP)-conjugated goat anti-mouse IgG (Sigma-Aldrich, St. Louis, MO, USA) diluted at 1:5000. The absorbance was measured at a wavelength of 450 nm (OD_450_), and titers defined as the highest dilution that yielded an OD_450_ value more than twice that of the pre-immune serum. To determine the subtype of FimA-specific IgG, serum samples diluted at 1:1000 were used as primary antibodies, and HRP-conjugated goat anti-mouse IgG1, IgG2a, IgG2b, and IgG3 (Proteintech, Wuhan, Hubei) used as secondary antibodies.

### Cytokine assays

One week after the final immunization, serum was harvested using a 1-mL syringe. Samples were centrifuged at 8,000 rpm for 5 min, and the supernatants collected and stored at −80 °C until analysis. Cytokine levels in the serum were determined using the Cytometric Bead Array-based Flow Cytomix assay (LEGENDplex Panel, Cat No. 740446; BioLegend, San Diego, CA, USA), according to the manufacturer’s instructions for the following proinflammatory cytokines: tumor necrosis factor (TNF)-α and interferon (IFN)-γ. The samples were thawed at room temperature prior to testing. All assays were performed in duplicate.

### Comparative efficacy of serum opsonophagocytic killing activity induced by FimA antigen with different adjuvants against hvKP YBQ *in vitro*

Immune sera collected from BALB/c mice immunized with the FimA antigen adjuvanted with different formulations were incubated at 56 °C for 30 min to inactivate the complement. YBQ was reactivated, and the bacterial concentration adjusted to 2.5 × 10^3^ CFU/mL using Oxoid brain-heart infusion broth (OBB). HL-60 cells were cultured to a specific density, adjusted to 4 × 10^5^ cells/mL in culture medium containing 0.8% dimethylformamide, and then induced to differentiate at 37 °C with 5% CO_2_ for four days. After differentiation, the cells were centrifuged at 350 × *g* for 5 min, the supernatant discarded, and the cells washed once with 1× Hank’s Balanced Salt Solution (HBSS; without Ca^2+^ and Mg^2+^), followed by 1× HBSS (with Ca^2+^ and Mg^2+^). The cells were resuspended in OBB, adjusted to a concentration of 1 × 10^7^ cells/mL, and then mixed with rabbit complement at a ratio of 4:1. In a 96-well cell culture plate, 20 µL OBB was added to each control well and 20 µL polyclonal antibodies to each test well, with 10 µL bacteria added to all wells. The plate was incubated at 37 °C at 700 r/min for 1 h, followed by the addition of 50 µL cell-complement mixture to each well and further incubation at 37 °C with 5% CO_2_ at 700 r/min for 1 h. After incubation, the 96-well plate was placed on ice for 20 min to terminate phagocytosis. Then, 5 µL of the mixture from each well (except for PBS, which was diluted five times in advance) was spread onto LB agar plates and incubated at 37 °C for 16–18 h. The number of surviving colonies on agar was counted to calculate the phagocytosis rate.

### Combined treatment with antibodies and antibiotics

Immune sera were obtained from mice immunized with Mix-epitope + AlPO_4_. Recipient mice received a single tail-vein injection of 100 μL immune or naive serum. Mice were intratracheally challenged with lethal or sublethal doses of YBQ. At 1 h after infection, the mice were randomly divided into three groups and subjected to the following treatments via the tail vein for immune serum and simultaneously administered with subcutaneous injections of meropenem: immune serum + meropenem (22.4 mg/kg, as described previously (27)), meropenem only (22.4 mg/kg), and a blank control. The survival rate was monitored daily for seven days, and bacterial colonization in the tissues quantified at 48 h after infection.

### Linear B-cell epitope mapping

To determine the reactivity of serum samples from immunized mice against each peptide, the wells of microtiter plates were coated with 5 µM of each peptide dissolved in 50 mM carbonate buffer (pH 9.6), and OVA_192–201_ peptide used as a negative control. Non-specific binding was prevented by blocking the coated microtiter plates with PBS (pH 7.4) containing 2% BSA. Serum samples from immunized mice (n = 10) were diluted in PBS at a ratio of 1:50 (v/v) and used as primary antibodies. Peroxidase-conjugated rabbit anti-mouse IgG antibodies (Solarbio, Beijing, China) were used as secondary antibodies at a dilution of 1:5000. The ELISA results are shown as OD_450_ values. The normal value for each peptide was calculated by testing sera from naive mice without immunization, and values >2.1-fold higher than the mean absorbance value of negative sera defined as positive.

### B-cell immunodominant epitope prediction

The prediction of B-cell immunodominant epitopes in FimA was predicted using the Immune Epitope Database and Analysis Resource (IEDB). Specifically, the Bepipred Linear Epitope Prediction tool under the “Linear B-Cell Epitope Prediction” module of IEDB was employed for the analysis. Default parameters of the tool were used throughout the analysis to ensure standardization and comparability of the prediction results.

### Structural localization and sequence alignment of the immunodominant epitopes

The crystal structure of FimA (PDB code: 6JZK) was obtained from the Protein Data Bank (PDB). Immunodominant epitopes were located on these structures using the PyMOL (v.1.1) program. FimA sequences from different KP strains were retrieved from the GenBank database for alignment using NCBI Basic Local Alignment Search Tool (BLAST) software.

### Statistical analysis

Statistical analyses were performed using GraphPad Prism (v.8.0; GraphPad Software, San Diego, CA, USA). All data are presented as means ± standard error of the mean (SEM). Data were analyzed using one-way analysis of variance with Bonferroni correction. A P-value <0.05 was considered statistically significant.

## Results

### Immunization with FimA in different adjuvant formulations protects against hvKP YBQ infection in a murine model of pneumonia

To evaluate the immunogenicity of FimA, BALB/c mice were intramuscularly immunized on days 0, 7, and 14 with recombinant FimA formulated with one of the three test adjuvants: AlPO_4_, AddaS03, or AddaVax. One week after the final immunization, mice were challenged with a lethal dose of the hvKP YBQ strain via tracheal intubation.

**Figure 1:**
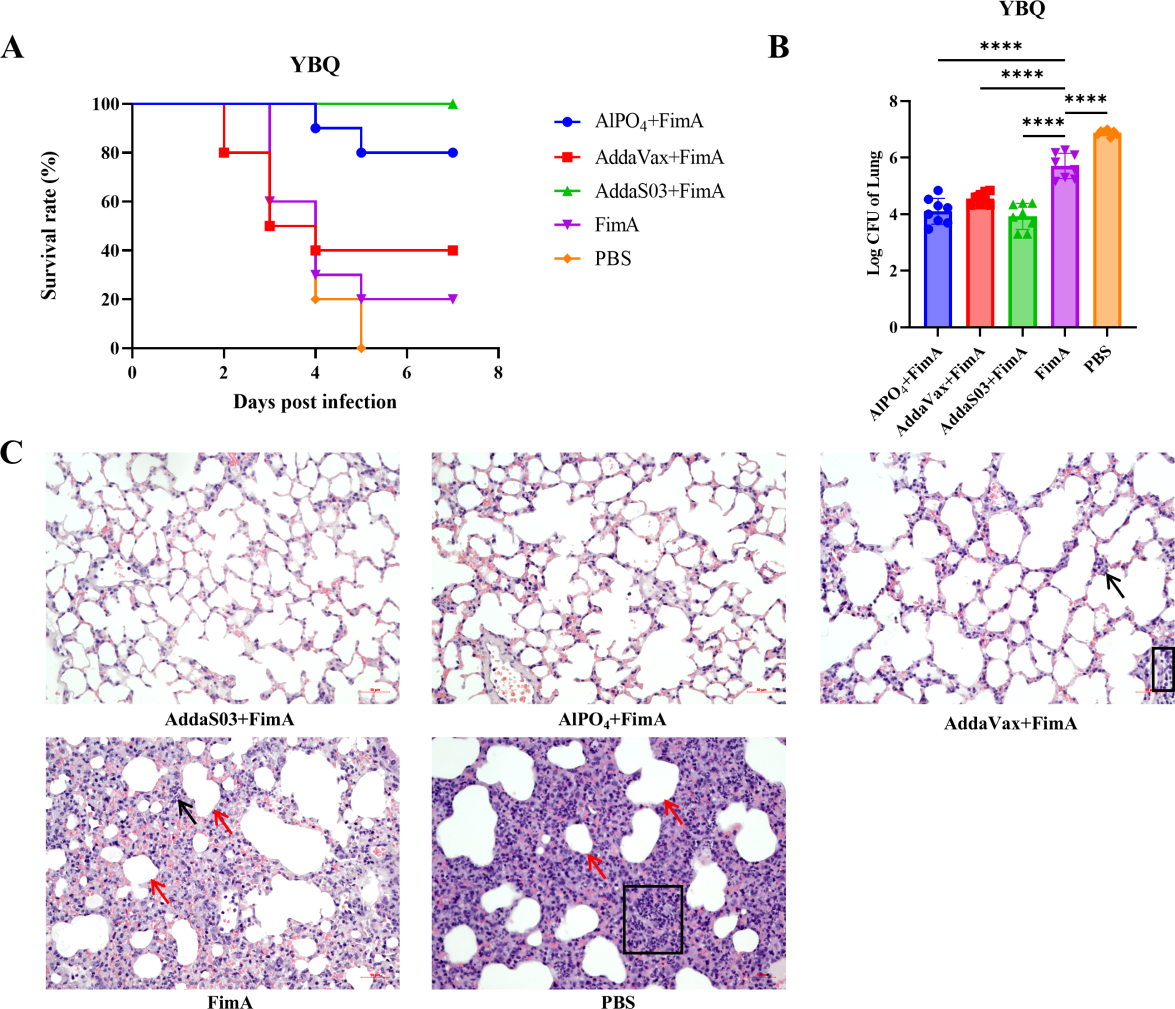
Protective efficacy of recombinant FimA formulated with different adjuvants against hvKP YBQ strain infections in an acute pneumonia murine model. BALB/c mice were intramuscularly immunized with recombinant FimA + PBS, PBS only, or FimA adjuvanted with AlPO_4_, AddaS03, or AddaVax (n = 8–10 mice per group); non-immunized mice served as controls. One week after the final immunization, all mice were challenged with lethal or sublethal doses of the hvKP YBQ strain via endotracheal intubation. ​ **(A)** Survival rates were monitored daily for seven days following lethal challenge. Data are presented as Kaplan–Meier curves, and statistical significance was analyzed using the log-rank test. **(B)** Bacterial loads in lung tissues were quantified 48 h after sublethal challenge. Data are expressed as log10-transformed colony-forming units (CFU)/g of tissue. **** indicates P <0.0001. **(C)** Representative histopathological images of lung sections at 48 h after sublethal challenge (H&E staining, original magnification: 200×). Black arrows indicate inflammatory cell infiltration, and red arrows denote alveolar structural damage.

As shown in **Figure 1A**, all mice in the PBS control group died within five days after infection, which was consistent with the results of our previous studies, confirming the successful establishment of an acute pneumonia model (16). Vaccination with FimA plus different adjuvants conferred varying degrees of protection. The FimA + AddaS03 group showed the highest survival (100%) with no clinical signs, followed by the FimA + AlPO_4_ (80% survival) and FimA + AddaVax (40% survival) groups. The FimA-only group exhibited a survival rate of only 20%. Statistical analyses using the Kaplan–Meier method revealed significantly improved survival in the FimA + AddaS03 (P = 0.0003) and FimA + AlPO_4_ (P = 0.0036) groups compared with that of the FimA-only group, whereas survival in the FimA + AddaVax group did not significantly differ (P = 0.7530). These results confirmed that FimA combined with specific adjuvants elicits strong and differential protection against hvKP challenge.

To assess the bacterial clearance ability induced by vaccination, mice immunized with FimA plus adjuvants were challenged with a sublethal dose of YBQ, and lung bacterial loads were subsequently quantified. As expected, all adjuvanted FimA groups showed a significantly lower pulmonary bacterial burden than that in the PBS control group (**Figure 1B**). Moreover, the FimA + AlPO_4_, FimA + AddaVax, and FimA + AddaS03 groups exhibited markedly reduced bacterial colonization compared with that in the FimA-only group (P <0.0001). Notably, the FimA + AddaS03 group had the lowest bacterial load among all the immunized groups.

Histopathological examination of lung tissues via H&E staining further supported the differential protective efficacy of the vaccine formulations (**Figure 1C**). The AddaS03 + FimA group showed the best preservation of lung architecture with minimal inflammatory infiltration; the AlPO_4_ + FimA group presented moderate inflammation and relatively intact alveoli; and in contrast, the AddaVax + FimA group displayed more severe inflammatory infiltration and partial alveolar damage. The FimA-only group showed an exacerbated pathology, whereas the PBS group exhibited the most severe damage, including extensive inflammatory infiltration and alveolar destruction.

### FimA immunization with different adjuvants differentially modulates humoral immune responses and cytokine profiles

Serum samples were collected from immunized BALB/c mice seven days after the final immunization to characterise the humoral immune responses induced by FimA that had been formulated with different adjuvants.

**Figure 2:**
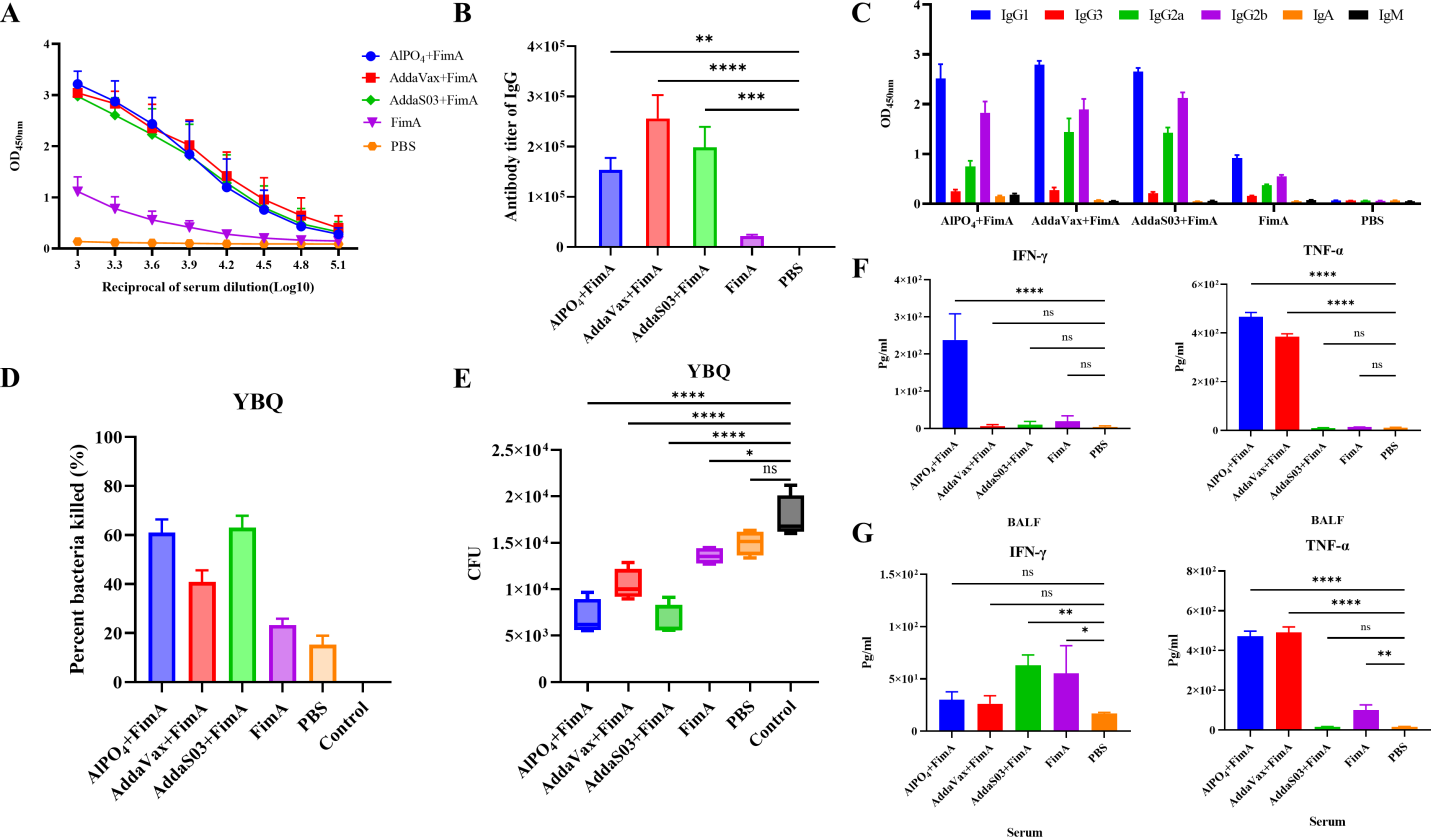
Adjuvant-dependent modulation of humoral and cellular immune responses in BALB/c mice immunized with FimA. BALB/c mice were intramuscularly immunized with recombinant FimA alone or formulated with AlPO_4_, AddaS03, or AddaVax (non-immunized mice served as controls; n = 8 per group). Sera and BALF were collected one week after the third immunization for subsequent analyses.​ **(A)** FimA-specific antibody reactivity was measured via ELISA. Data are shown as OD_450_ values across serial serum dilutions (mean ± SEM).​ **(B)** Endpoint titers of FimA-specific IgG. Antibody titer was defined as the highest serum dilution at which the OD_450_ value was ≥0.2 (cut-off value); data are presented as mean ± SEM of the titers in each group.​ **(C)** IgG subclass profiles of the FimA-specific antibodies. Relative levels of FimA-specific IgG subtypes (e.g. IgG1, IgG2b, IgG2a, IgG3, IgA, and IgM) in serum were determined via subtype-specific ELISA; results are expressed as mean OD_450_ values ± SEM.​ **(D)** OPK efficiency (%) results. Data represent the percentage of bacteria killed relative to those in the non-immunized control group (mean ± SEM).​ **(E)** Bacterial load in the OPK assay. Surviving bacteria in the OPK assay were enumerated through colony counting; results are presented as log_10_ CFU/well (mean ± SEM).​ **(F, G)** Concentrations of IFN-γ and TNF-α in **(F)** BALF and **(G)** serum; data are expressed as pg/mL (mean ± SEM).​ Significant differences are indicated as *P <0.05, **P <0.01, ***P <0.001, and ****P <0.0001; “ns” indicates no significant difference.

FimA immunization in combination with different adjuvants elicited varying levels of FimA-specific IgG antibodies. As shown in **Figures 2A, B**, the adjuvant-formulated groups exhibited significantly higher antibody titers than the FimA-only group did. The mean titers were 153,600 for FimA + AlPO_4_ (P = 0.0027), 256,000 for FimA + AddaVax (P <0.0001), and 198,400 for FimA + AddaS03 (P = 0.0001). In contrast, the FimA-only group showed a markedly lower titer (21,600), which did not significantly differ from that of the PBS control group (P = 0.9669). Analysis of the antibody subclasses revealed a consistent pattern across all adjuvanted groups: IgG1 was the dominant subtype, followed by IgG2b and IgG2a, suggesting a Th2-skewed humoral response (**Figure 2C**).

The functional activity of FimA-specific antibodies was assessed using an *in vitro* opsonophagocytic killing (OPK) assay against strain YBQ. As illustrated in **Figure 2D**, the bactericidal rates were 15.3% in the PBS control group and 23.3% in the FimA-only group. In contrast, a significantly higher OPK activity was observed in the adjuvanted groups: 60.9% for FimA + AlPO_4_, 40.9% for FimA + AddaVax, and 63.0% for FimA + AddaS03. Consistent with these findings, quantification of the remaining bacterial load confirmed the same trends (**Figure 2E**). These results align with the survival and bacterial colonization data, underscoring the critical influence adjuvants have in enhancing the functional efficacy of FimA-induced antibodies.

Cytokine profiles in bronchoalveolar lavage fluid (BALF; reflecting local immunity) and serum (reflecting systemic immunity) were evaluated to assess adjuvant-driven cellular immune responses. In BALF, the AlPO_4_ group showed elevated IFN-γ levels, whereas both AddaVax and AlPO_4_ groups had increased TNF-α levels (P <0.05). In serum, higher IFN-γ levels were detected in the AddaS03 and FimA-only groups, and elevated TNF-α levels were observed in the AddaVax, AlPO_4_, and FimA-only groups compared with those in the PBS control group (P <0.05). Notably, AddaS03 elicited a selective increase in systemic IFN-γ expression, without inducing substantial TNF-α release, implicating a potential role for this Th1 cytokine in supporting humoral immunity. In contrast, AlPO_4_ primarily induced a local Th1 response and AddaVax showed weak Th1 induction, consistent with their respective protective efficacies. As cytokines were assessed prior to challenge, they reflect vaccine-induced immune activation and may not directly indicate protective immunity.

### Identification and adjuvant-mediated modulation of immunodominant B-cell epitopes on FimA

To elucidate the molecular mechanisms underlying the adjuvant-driven differences in FimA-specific immunity, we mapped immunodominant linear B-cell epitopes using antisera collected on day 7 after the final immunization from mice immunized with FimA that had been formulated with AddaS03, AlPO_4_, or AddaVax. Epitope screening was performed using an overlapping peptide ELISA approach, in which a library of 25 synthetic peptides (each 18-amino-acids long with a 12-amino-acid overlap) tiling the full-length FimA protein was employed. Three peptides were identified as immunodominant based on peptide-specific IgG reactivity: FimA_73–90_ (ANEAPITNVLALDAGNPT), FimA_97–114_ (KLTDRNNTPVTLDKPFDP), and FimA_103–120_ (NTPVTLDKPFDPNVDPRI) (**Table 1**). The nomenclature of each epitope reflects its positional indices within the FimA sequence.

**Table 1:** Sequences of the immunodominant epitopes on the FimA identified in this study.

The immunodominant epitope	Adjuvant	Sequence of the immunodominant epitope
FimA_73-90_	AddaVax, AddaS03	ANEAPITNVLALDAGNPT
FimA_97-114_	AddaVax, AlPO_4_	KLTDRNNTPVTLDKPFDP
FimA_103-120_	AddaVax, AddaS03, AlPO_4_	NTPVTLDKPFDPNVDPRI

**Figure 3:**
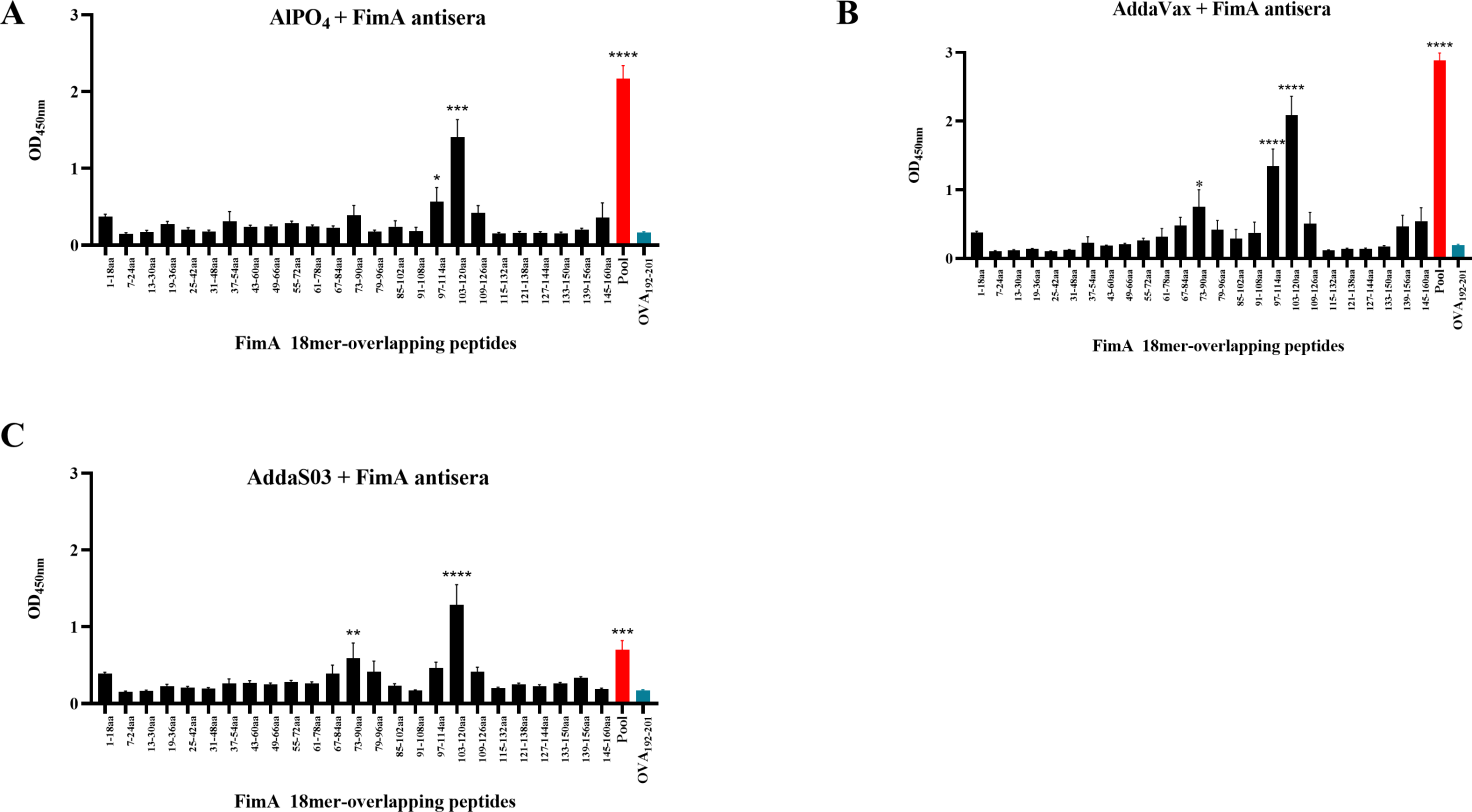
Identification of B-cell immunodominant epitopes of FimA using immune sera obtained from mice immunized with FimA plus different adjuvants​. Immune sera were collected from BALB/c mice one week after the third intramuscular immunization with FimA + AlPO_4_, FimA + AddaVax, or FimA + AddaS03. B-cell epitopes of FimA were identified via overlapping peptide ELISA; a panel of synthetic overlapping peptides was coated onto ELISA plates, and serum reactivity to each peptide then detected. The peptide pools served as positive controls, and OVA_192–201_ was used as a negative control. **(A–C)** Serum reactivity profiles of the **(A)** FimA + AlPO_4_, **(B)** FimA + AddaVax, and **​(C)** FimA + AddaS03 groups. Data represent the mean OD_450_ values ± SEM of serum reactivity to each FimA-overlapping peptide. Peptides with OD_450_ values significantly higher than those of the negative control (set as 2 × OD_450_ of the non-immunized serum) were defined as immunodominant epitopes.​ Significant differences are indicated as *P <0.05, **P <0.01, ***P <0.001, and ****P <0.0001.

Notably, the immunodominance hierarchy of these epitopes was obviously associated with the adjuvant used. As summarized in **Figure 3A**, AddaS03 was associated with strong responses of FimA_73–90_ and FimA_103–120_. In the AlPO_4_-adjuvanted group (**Figure 3B**), FimA_97–114_ and FimA_103–120_ were immunodominant. In contrast, all three epitopes were predominantly recognized in the AddaVax group (**Figure 3C**). The reactivity signals of these peptides were statistically significant, supporting their identification as immunodominant epitopes under the experimental conditions used. A search of the IEDB and related literature indicated that these three epitopes have not been previously reported in the context of KP FimA, suggesting their potential novelty. These results suggest an association between adjuvant formulation and epitope immunodominance patterns and may provide useful information for the rational selection of epitopes for future FimA-based subunit vaccines.

### Localization and sequence conservation of immunodominant epitopes on FimA

**Figure 4:**
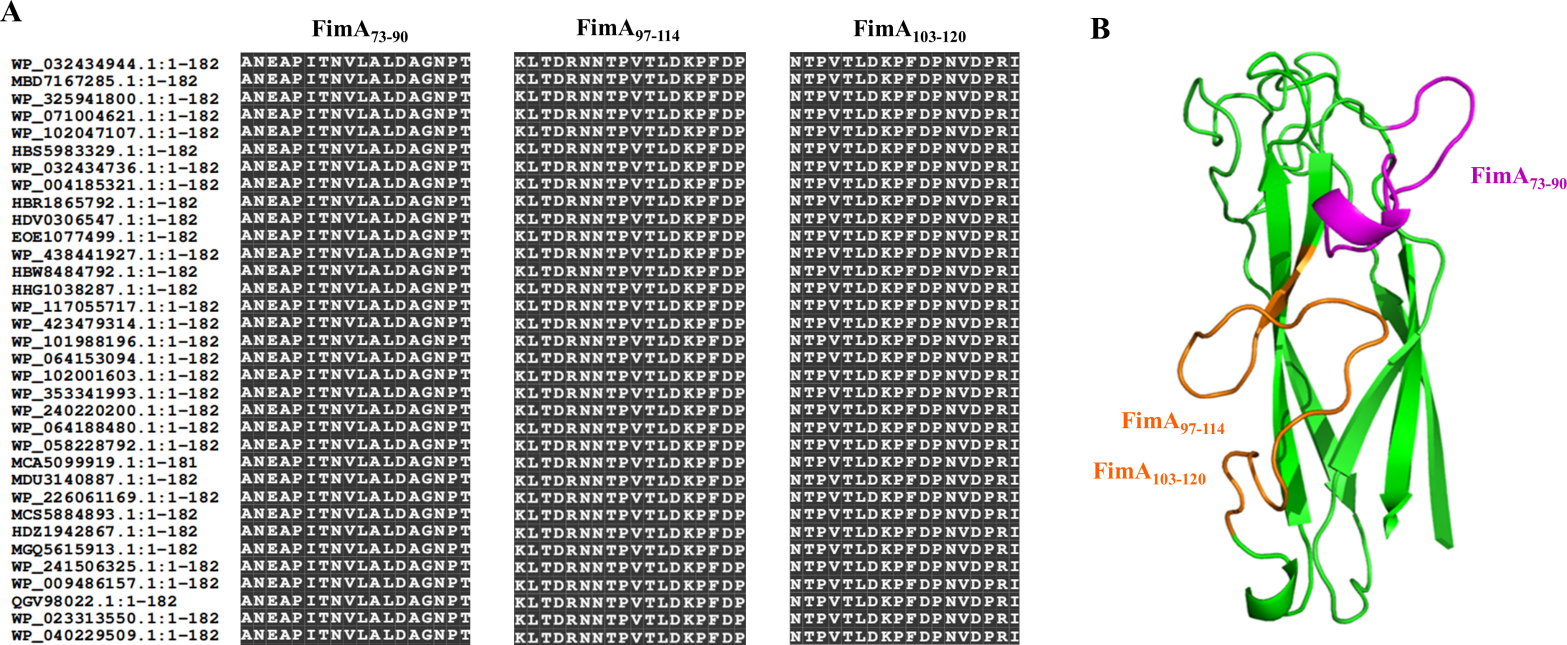
Conservation analysis and spatial localization of the FimA B-cell immunodominant epitopes. Analyses were performed using the three novel FimA B-cell immunodominant epitopes identified in this study.​ **(A)** Sequence conservation of epitopes across 34 KP strains detected via BLAST analysis; the KP strains were selected from the NCBI GenBank database. Amino acid sequences of the three epitopes were aligned with the corresponding regions of FimA from the 34 KP strains using BLASTp. **(B)** Spatial localization of epitopes in the FimA 3D structure determined via PyMOL. The 3D structure of FimA was retrieved from the Protein Data Bank (PDB ID: 6JZK), onto which the three immunodominant epitopes were mapped using PyMOL.

To evaluate the conservation of the identified immunodominant epitopes, we performed a sequence alignment of FimA proteins derived from 34 randomly selected KP strains obtained from the GenBank database. The alignment revealed that all three epitopes were completely conserved across all 34 strains, displaying 100% amino acid identity (**Figure 4A**). This high degree of conservation suggests that antibodies targeting these epitopes exhibit broad cross-reactivity against diverse KP strains, supporting their potential as universal components in a KP vaccine.

To explore the structural basis of the observed differences in immunodominance hierarchy and their correlation with protective efficacy, we mapped the three epitopes onto the crystal structure of FimA (PDB ID: 6JZK) using PyMOL software. As illustrated in **Figure 4B**, all immunodominant epitopes were situated on the protein surface, rendering them accessible to antibody-binding. However, they displayed distinct structural properties. FimA_73–90_ adopts a well-defined secondary structure comprising a short α-helix and flexible loop (β-turn), which forms a stable, surface-exposed structural motif ideal for high-affinity antibody binding. In contrast, FimA_97–114_ and FimA_103–120_ are predominantly flexible random coils without stable secondary folding. Notably, despite their high conformational flexibility, their full surface accessibility and ability to elicit robust linear antibody responses likely underpin the superior immunogenicity and protective efficacy of FimA_103–120_ observed in our immunization studies.

### Immunization with a mixture of FimA immunodominant epitopes conjugated to KLH confers protection against hvKP challenge

**Figure 5:**
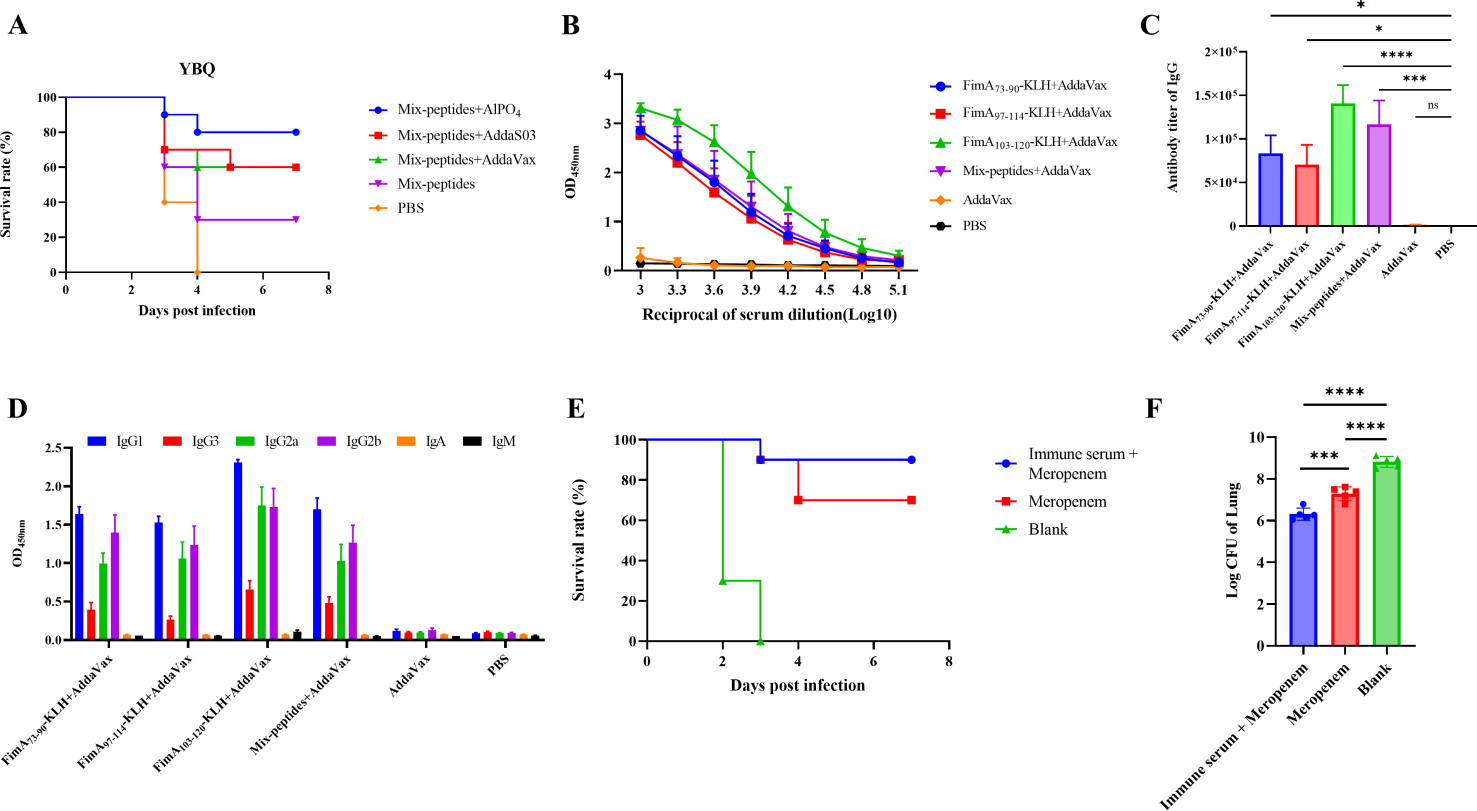
Protective and therapeutic efficacy analyses of the three FimA B-cell immunodominant epitopes. BALB/c mice were intramuscularly immunized with individual immunodominant epitope–KLH peptide + AddaVax; mixed immunodominant epitope–KLH peptides (equal ratio of the three individual epitopes) + AddaVax, AddaS03, or AlPO_4_; or AddaVax + PBS or PBS only (n = 10 per group). Mice received three immunizations at 1-week intervals; sera were collected one week after the third immunization, and all groups challenged with a lethal dose of the hvKP YBQ strain via endotracheal intubation one week after the final immunization. **(A)** Survival rates of mice monitored daily for seven days after the hvKP YBQ strain challenge. Data are presented as Kaplan–Meier survival curves.​ **(B)** Epitope-specific antibody OD values. Serum reactivity to each corresponding epitope was detected via ELISA using serial serum dilutions. Data are presented as mean OD_450_ values ± SEM across the dilutions, with each group represented by distinct curves.​ **(C)** Epitope-specific antibody titers. Antibody titer was defined as the highest serum dilution with an OD_450_ value ≥0.2 (cut-off value). Data are presented as mean titer values ± SEM, with each bar representing a group to enable direct comparisons.​ **(D)** Epitope-specific antibody subtypes. Relative levels of IgG1, IgG2a, IgG2b, IgG3, IgA, and IgM against each epitope or the mixed epitope pool were measured using subtype-specific ELISA. Data are expressed as mean OD_450_ values ± SEM. **(E)** Survival rates of mice measured after the hvKP YBQ strain challenge. Mice were treated with immune serum derived from Mix-peptide-immunized mice plus meropenem, meropenem alone, or naive serum (blank control). Survival status was monitored daily for seven days after hvKP challenge, and data plotted as Kaplan–Meier survival curves. **(F)** Bacterial loads in the lung tissues of mice after sublethal challenge with the hvKP YBQ strain. The corresponding treatment groups are consistent with those described in **(E)**. Lung tissues were collected 48 h after hvKP challenge, and bacterial loads were quantified and expressed as log_10_-transformed CFU/g of tissue. Significant differences are indicated as **P <0.01, ***P <0.001, and ****P <0.0001; “ns” indicates no significant difference.

To evaluate the protective potential of the identified immunodominant epitopes, we prepared Mix-peptides consisting of FimA_73–90_–KLH, FimA_97–114_–KLH, and FimA_103–120_–KLH, each conjugated to KLH as a carrier protein. Mice were intramuscularly immunized on days 0, 7, and 14 with the Mix-peptides formulated with AlPO_4_, AddaVax, or AddaS03, and survival was monitored following challenge with a lethal dose of the hvKP YBQ strain on day 7 after final immunization. As shown in **Figure 5A**, the Mix-peptides + AlPO_4_ group conferred 80% survival, whereas both the Mix-peptides + AddaVax and Mix-peptides + AddaS03 groups achieved 60% survival. In contrast, only 30% of mice that had received Mix-peptides alone survived, which was still significantly higher than that of the PBS control group (P <0.01). These data indicated that epitope-based vaccines can elicit protective immunity, which is further enhanced by adjuvantation.

To further characterize the immunogenicity of each epitope, we immunized mice with individual KLH-conjugated immunodominant epitopes formulated with AddaVax, which was selected to ensure consistent comparative analyses, and measured epitope-specific antibody responses. As summarized in **Figures 5B, C**, all immunized groups showed statistically significant differences in antibody titers compared with those in the PBS group. The FimA_103–120_–KLH + AddaVax group exhibited the highest mean titer up to 140,800, suggesting that FimA_103–120_ is the most immunogenic of the three epitopes tested.

Antibody subclass analysis revealed a consistent Th2-skewed response across all epitope-immunized groups, dominated by IgG1, followed by IgG2b and IgG2a (**Figure 5D**). This profile mirrors that induced by the full-length FimA protein (Section 3.2), supporting the conclusion that both the whole protein and its dominant epitopes preferentially drive Th2-polarized humoral immunity when delivered with adjuvants such as AddaVax.

To assess the therapeutic potential of serum induced by the immunodominant epitopes, we conducted a passive immunization experiment. Survival analysis (**Figure 5E**) showed that mice treated with serum from the Mix-peptides + AlPO_4_ group in combination with a low dose of meropenem achieved the highest survival rate of 90%, followed by the meropenem monotherapy group (70% survival rate), whereas all animals in the naive serum control group died of infection. Notably, statistical analyses verified that the survival rates of both the Mix-peptides + AlPO_4_ serum and meropenem groups were significantly higher than those in the PBS control group (P <0.0001). To dissect the underlying mechanism responsible for this therapeutic efficacy, we further quantified bacterial colonization levels in the lungs of challenged mice (**Figure 5F**), which revealed that mice receiving Mix-peptides + AlPO_4_-immunized serum with a low dose of meropenem exhibited the lowest pulmonary bacterial loads, with CFU counts reduced by approximately 1–2 orders of magnitude compared with those in the PBS control group (P <0.0001); consistent with this, the meropenem group displayed a marked decrease in bacterial colonization (P <0.0001). Notably, the CFU counts in the combination therapy group were significantly lower than those in the meropenem alone group (P = 0.0005), corresponding to an approximate 1-log reduction in bacterial burden.

Collectively, these findings demonstrate that serum induced by FimA immunodominant epitopes with sub-therapeutic antibiotic dosing exerts robust therapeutic effects against hvKP challenge, which is closely correlated with its capacity to suppress bacterial colonization in this key target organ, this synergistic effect arises from FimA-specific immune serum promoting opsonophagocytosis and inhibiting bacterial adhesion to enhance antibiotic access to bacterial niches, thus highlighting the promising potential of these epitopes as dual-purpose candidates for both prophylactic and therapeutic vaccine development.

## Discussion

KP infections exert a significant global public health and financial burden because it can cause severe pneumonia, which has high mortality rates owing to the rise in antimicrobial resistance rates (28). Regional surveillance data underscore the scale of this issue; in Southern Europe, CRKP has been detected at a notably high rate (62.8%) among bloodstream infections (29). In Asia, the spread and drug resistance of KP are growing concerns, with a systematic review estimating the pooled prevalence of nosocomial MDR KP to be 32.8% (30). Similarly, KP represents a major public health threat in the Americas, where the increased prevalence of hypervirulent CRKP has been linked to the COVID-19 pandemic (31). Some virulent CRKP strains have been documented to share certain hypervirulent phenotypic and molecular features with classic hvKP strains, a characteristic that is also exhibited by the hvKP strain YBQ and has been confirmed in clinical CRKP ST392 isolates in relevant phenotypic and molecular studies (32). These trends highlight the escalating difficulty in managing KP infections worldwide and underscore the urgent need to develop effective preventative strategies, such as vaccines, and therapeutic approaches, including antibody–antibiotic combinations, to combat this pathogen (33).

YBQ, a clinical hvKP isolate exhibiting a hypermucoviscous phenotype with demonstrated high pathogenicity (26), was exclusively used as the hvKP challenge strain in this study to validate the protective efficacy of FimA-based immunization strategies. It shares key phenotypic and genetic characteristics with the currently prevalent KP clones—such as the globally emerging hypervirulent ST23 clone, which is dominant in China and carries the virulence genes, *rmpA* and *iucA*, on horizontally transmissible IncFIB plasmids (34), as well as high-risk hypervirulent ST65/ST86 clones associated with the KL64 capsular type and aerobactin-mediated iron acquisition (35). Based on these shared attributes, we propose that YBQ serves as an hvKP strain that can be used for the development of novel FimA protein-based protective strategies against prevalent KP infections. The findings obtained from this isolate are likely generalizable to clinically relevant and widely circulating clones. Relevant information on this strain is presented in the **Supplementary Materials** (**Supplementary Figures 1A–D**).

The selection of appropriate antigens is critical for developing effective vaccines against KP. A range of vaccine candidates have previously been explored, including whole bacterial lysates, outer membrane proteins, lipopolysaccharides, and fimbrial antigens (12). An ideal antigen should be widely conserved across serotypes, readily accessible to the immune system, and capable of eliciting protective immunity. A systematic review of human vaccine candidates for KP infections highlighted recombinant antigen-based vaccines as a promising strategy, owing to their high conservation and ability to be recognized by the host immune system. In this context, FimA, a key pilus protein in KP, is a promising antigen candidate for preventing KP infections (33). Although Mrk fimbriae are important for biofilm formation and medical device-related infection (36), FimA mediates critical mannose-dependent adhesion to host mucosal epithelia, which represents an early and essential step in both mucosal and systemic KP infection. Furthermore, FimA exhibits high conservation across different isolates and low cross-reactivity with commensal enterobacteria (**Supplementary Figures 2A, B**), making it superior to many serotype-dependent or highly conserved outer membrane proteins (37) as a broad-spectrum vaccine target. A previous study demonstrated that a trivalent vaccine incorporating FimA conferred protection in a murine pneumonia model of KP infection (16). In the present study, recombinant FimA acted as an effective protective antigen that elicited robust antibody-dependent immunity against infection with the hvKP YBQ strain. By formulating FimA with distinct adjuvants, we not only confirmed its efficacy against an hvKP strain but also demonstrated that the magnitude and functional quality of the FimA-specific antibody response, and consequently the level of protection, are critically shaped by the accompanying adjuvant.

Antibody responses serve as primary indicators of adaptive immunity against KP, with critical parameters, such as antigen dose, immunization route, and schedule, often tailored to specific disease models, vaccine platforms, and intended-use settings (33). In this study, sera collected from mice immunized with recombinant FimA combined with different adjuvants demonstrated significant functional activity against the hvKP YBQ strain. We observed notable differences in OPK activity when using immune serum antibodies derived from groups immunized with FimA that had been formulated with different adjuvants. This variability in functional antibody activity likely explains the differences observed in protective immunity elicited by each adjuvant formulation, reinforcing the established principle that adjuvants substantially enhance both the magnitude and functional quality of antibody responses. Serum antibody subtyping across all FimA–adjuvant groups consistently revealed a predominance of IgG1, followed by IgG2b, suggesting a polarized immune bias. Crucially, our findings demonstrated that the protective efficacy of a FimA-based vaccine is highly dependent on the selected adjuvant, underscoring that adjuvant choice is a fundamental determinant of immunogenicity and overall vaccine effectiveness. This conclusion is supported by broader vaccine development efforts against KP, which highlight the necessity of rational adjuvant selection to optimize protective immunity (38). Building on the protective potential of FimA-specific antibodies, we further explored their therapeutic value in combination with antibiotics. Our preliminary data demonstrated that immune serum collected from epitope cocktail-immunized mice, when co-administered with sub-therapeutic doses of meropenem, not only improved survival rates in hvKP-challenged mice but also significantly reduced bacterial loads in lung tissues. This synergistic effect holds particular significance in the context of global antimicrobial resistance, and antibody-mediated opsonization enhances the phagocytic clearance of hvKP, thereby lowering the required antibiotic dosage and mitigating selective pressures that drive the emergence and spread of drug-resistant strains (39). This synergistic antibacterial mechanism is consistent with our previous findings in *Staphylococcus aureus* (40).

Notably, total IgG titer did not positively correlate with protective efficacy, as AddaVax induced the highest antibody titer but conferred the lowest survival rate. This discrepancy indicates that antibody functional quality, rather than mere quantity, determines protection against KP. A balanced Th1/Th2 response and robust OPK activity, rather than high total IgG levels, are critical for effective bacterial clearance and host survival. Among the three adjuvants evaluated in this study, AddaS03 demonstrated the strongest protective efficacy against the clinical hvKP YBQ strain. Antibody isotype analysis and cytokine profiling revealed that AddaS03 induced a mixed Th1/Th2 immune response characterized by dominant Th2-associated IgG1, together with detectable Th1-associated IgG2a/IgG2b, and moderate levels of Th1-type cytokines. As measurements were performed prior to pathogen challenge, these data reflect the baseline immune activation state induced by different vaccine formulations, rather than the immune response elicited by bacterial infection itself. Therefore, these pre-challenge immune parameters should be interpreted as indicators of vaccine-induced immune activation and may not directly indicate protective immunity. In contrast, AlPO_4_ and AddaVax elicited comparatively weaker Th1 responses. The balanced Th1/Th2 polarization induced by AddaS03 is likely the key mechanism underlying its superior protective effects, as Th1 immunity promotes bacterial clearance through phagocyte activation and enhances OPK, whereas Th2 immunity facilitates long-term humoral protection via high-affinity antibody production. These findings are consistent with those of a previous report (41). However, because bacterial challenge was performed 7 days after the final immunization, the relatively short interval may not fully exclude the contribution of residual adjuvant-induced innate immune activation to the observed protection. Thus, the protective efficacy of different formulations may reflect both antigen-specific adaptive immune responses and, to some extent, adjuvant-associated innate immune activation. Future studies using extended intervals between the final immunization and bacterial challenge will be carried out to further exclude, if present, the role of adjuvant-induced innate immunity in the protective efficacy of different formulations. Although AS03 (a licensed adjuvant system similar to AddaS03) and MF59 (a squalene-based emulsion analogous to AddaVax) have been approved for influenza vaccines, our study highlights the potential of using AddaS03 and AddaVax as adjuvants in bacterial vaccine design. Thus, this study provides important insights into the development of novel adjuvant-based vaccines against a broad range of pathogens.

Immunodominant epitopes play a critical role in directing immune responses against pathogens because they represent primary targets recognized by the immune system during infection or vaccination. These epitopes may contribute to the specificity and focus of immune responses, and are often associated with the breadth and potency of the resulting immunity (42, 43). Consequently, the identification and characterization of immunodominant epitopes can provide useful information for rational vaccine development. Recent studies have demonstrated that vaccines targeting antibiotic-resistant bacteria can be engineered around such epitopes to induce protective immunity (44). In the present study, we identified three B-cell immunodominant epitopes on FimA, including three novel ones (FimA_73–90_, FimA_97–114_, and FimA_103–120_) whose immunodominance was affected by the adjuvant used. This finding is valuable because it suggests that adjuvants may be associated with differences in the antigenic regions targeted by the immune system, which can affect the breadth and effectiveness of immune responses. Furthermore, analogous observations have been reported in *S. aureus*, a Gram-positive bacterium (44), *Plasmodium vivax* (45) and *Helicobacter pylori* (46) indicating that adjuvant-driven modulation of epitope immunodominance is not restricted to Gram-negative pathogens, such as KP, but rather represents a conserved immunomodulatory mechanism with broader implications for pathogen vaccine design.

In this study, we used the clinical hvKP YBQ strain, which shares high sequence identity with predominant clinical isolates identified in China. The three immunodominant FimA epitopes identified were 100% conserved at the amino acid level across diverse KP strains, including globally prevalent isolates. This broad conservation highlights the potential of using these epitopes as targets for a broadly effective vaccine. Notably, among these epitopes, FimA_103–120_ induced a significantly higher antigen-specific antibody titer than those of FimA_73–90_ and FimA_97–114_. This enhanced immunogenicity may be attributed to its full surface accessibility and high conformational flexibility, which enable efficient recognition by B-cell receptors and robust elicitation of linear antibody responses. These findings help explain the observed hierarchy of immunodominance and its correlation with the varying protective efficacies elicited by different adjuvants, clarifying why the AddaS03-adjuvanted group achieved the highest survival rate. Therefore, monoclonal antibodies (mAbs) targeting the identified immunodominant epitopes of FimA could potentially serve as effective and specific therapeutic antibodies against KP infections (47). Considering the conserved sequence identity of the immunodominant epitopes of FimA across globally prevalent hvKP strains and clinical isolates obtained from China, the three novel immunodominant epitopes characterized in this study hold potential as broad-spectrum epitope-based vaccine candidates targeting the FimA antigen in KP infections. Additionally, given the specificity of mAbs, they can be used for passive immunization against hvKP infections to detect KP antigens in clinical samples, enabling a theranostic approach to KP management (48). Furthermore, we compared our experimentally identified epitopes with in silico predictions generated using linear B-cell epitope prediction tools available on the IEDB website. As summarized in **Table 2**, the computationally predicted epitopes did not fully align with our empirical findings. This discrepancy underscores that even in the era of advanced artificial intelligence tools, experimental validation remains crucial for confirming the authenticity and functional relevance of putative epitopes (49).

**Table 2:** Prediction results of B-cell immunodominant epitopes in FimA protein.

The immunodominant epitope	Sequence of the immunodominant epitope
FimA_18-24_	LASGNEK
FimA_71-77_	ASANEAP
FimA_86-93_	AGNPTAKK
FimA_99-129_	TDRNNTPVTLDKPFDPNVDPRITVNADGTGT
FimA_139-152_	WDKDNAEAGDGNAT

In conclusion, this study suggests that adjuvant selection is associated with differences in FimA-mediated protective immunity against KP. We identified three novel linear B-cell immunodominant epitopes on FimA named FimA_73–90_, FimA_97–114_, and FimA_103–120_, and found that their immunodominance hierarchy was associated with the adjuvant used in vaccination. This epitope-level divergence may be related to the observed differences in protective efficacy among the adjuvant groups. Our findings highlighted a potential role for adjuvants in changing the specificity, magnitude, and functional quality of FimA-directed immune responses, which may be linked to differences in vaccine efficacy. These results provide a rational basis for selecting adjuvants for FimA-based KP vaccine development and offer broader insights into epitope-focused vaccine design against other bacterial pathogens. In the future, preparing a group of mAbs that can target the three novel immunodominant epitopes of FimA could offer both therapeutic and diagnostic functions against KP infections. Additionally, exploring the synergistic efficacy of these mAbs with various antibiotics in clinical isolates of CRKP would provide a more comprehensive strategy for combating MDR pathogens.

## Data Availability

The original contributions presented in the study are included in the article/**Supplementary Material**. Further inquiries can be directed to the corresponding authors.
