# Supplementary material for: Adjuvant-driven epitope hierarchy correlates with the protective efficacy of FimA vaccine against Klebsiella pneumoniae
Source: Front Immunol. 2026 Jun 22;17:1796753. doi: 10.3389/fimmu.2026.1796753 (PMC13333469; doi:10.3389/fimmu.2026.1796753)
Supplement: Supplementary file 1 [file Table1.docx]

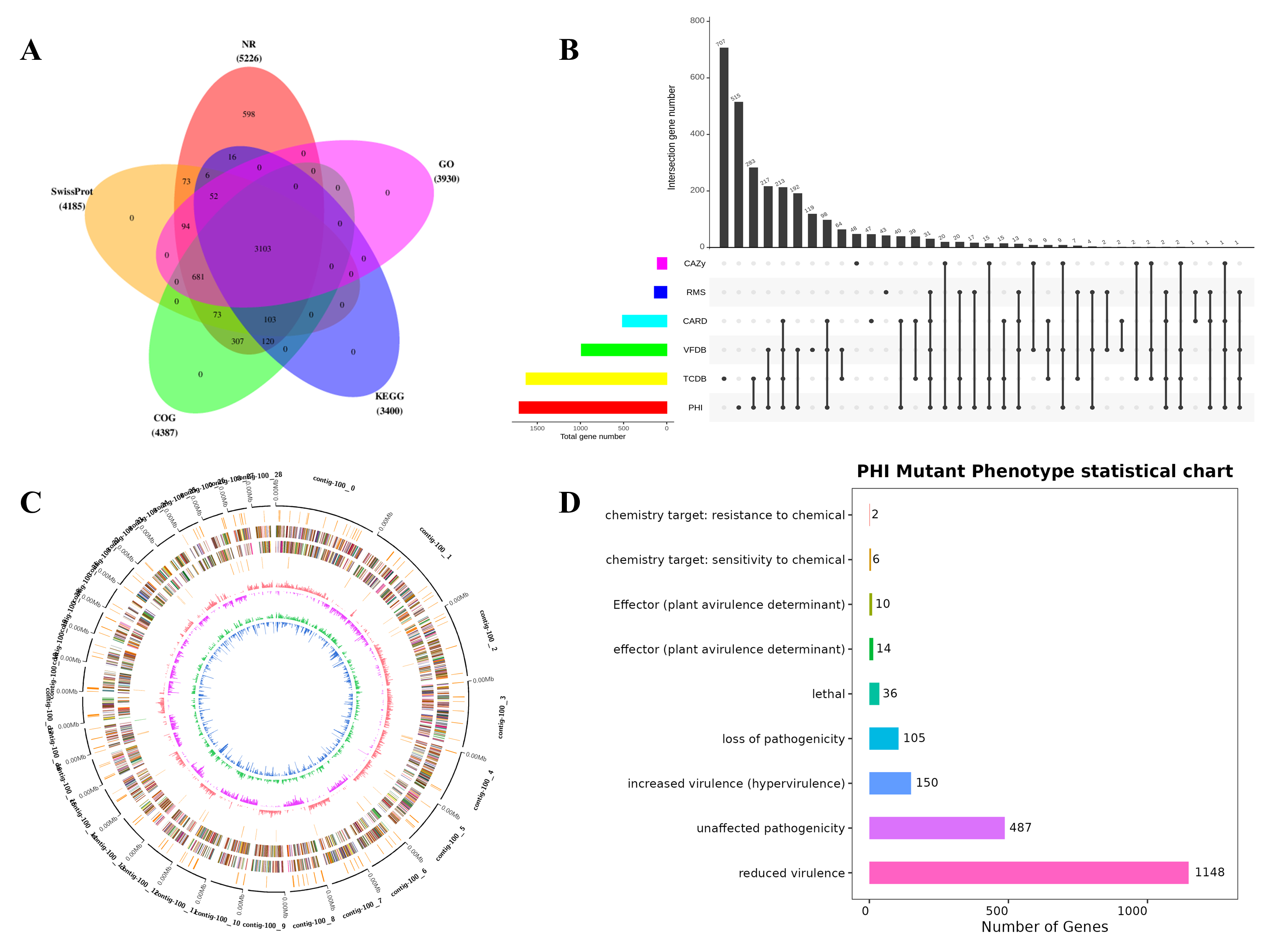


**Supplementary Figure S1. Genomic and functional profiles of hypervirulent *Klebsiella pneumoniae* YBQ**

(A) Shared and unique annotations in basic databases (NR, SwissProt, COG, KEGG, GO), showing overall sequence conservation. (B) Shared and unique annotations in specific functional databases (VFDB, PHI, TCDB, etc.), reflecting functional specificity. (C) Bacterial genome circular map, displaying GC content (56.36%), gene distribution and functional classification. (D) PHI mutant phenotype statistics, indicating pathogen-host interaction and pathogenicity-related traits.


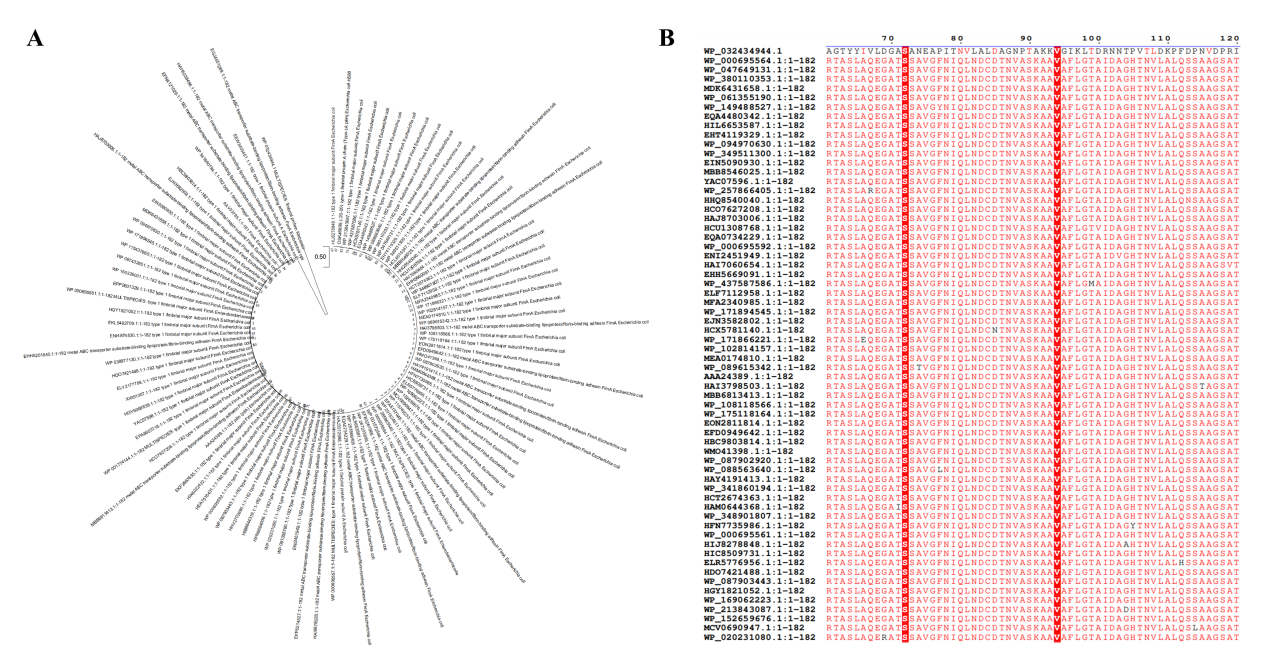


**Supplementary Figure S2. Phylogenetic relationship and sequence conservation of FimA between *Klebsiella pneumoniae* and commensal *Escherichia coli*.**

(A) Phylogenetic analysis of FimA proteins from KP and commensal *E. coli*. (B) Sequence alignment of FimA from KP and commensal *E. coli*.
